# The induction of oocyte maturation and ovulation in the European eel (*Anguilla anguilla*): *in vitro* and *in vivo* comparison of progesterone with 17α,20β-dihydroxy-4-pregnen-3-one

**DOI:** 10.3389/fphys.2023.1207542

**Published:** 2023-08-08

**Authors:** Pauline Jéhannet, Arjan P. Palstra, Miriam Meijerhof, Henk Schipper, Ignacio Nebot Giménez, Ron P. Dirks, William Swinkels, Leon T. N. Heinsbroek, Hans Komen

**Affiliations:** ^1^ Animal Breeding and Genomics, Wageningen University & Research, Wageningen, Netherlands; ^2^ Experimental Zoology, Wageningen University & Research, Wageningen, Netherlands; ^3^ Rara Avis, Valencia, Spain; ^4^ Future Genomics Technologies B.V., Leiden, Netherlands; ^5^ Palingkwekerij Koolen B.V., Bergeijk, Netherlands; ^6^ Wageningen Eel Reproduction Experts B.V., Wageningen, Netherlands

**Keywords:** aquaculture, eel reproduction, spawning induction, maturation-inducing hormone, RNA-seq

## Abstract

Ovulation in European eel is induced by injection of 17α,20β-dihydroxy-4-pregnen-3-one (DHP) as the maturation-inducing hormone (MIH). Female eels need to ovulate within 18 h after injection to release good quality eggs. Progesterone (P), as an upstream precursor of DHP, may promote endogenous DHP production and improve egg quality. The purpose of this study was therefore to compare treatment of P with DHP on batch level, *in vitro*, to determine dose-response effects, and *in vivo*, at a single dose. For the *in vitro* experiment, ovarian tissue was extracted and placed in culture plates containing hormone-free medium and media supplemented with the treatment: DHP at 1, 10 and 100 ng mL^−1^, or P at 10, 100 and 1,000 ng mL^−1^. At the start of incubation, the folliculated oocytes were sampled for histology, microscopy and qPCR. After incubation for 12 and 18 h, the oocytes were sampled for microscopy and qPCR analysis. For the *in vivo* experiment, females were either injected with DHP or P at a dose of 2 mg kg^−1^ to assess their effects on ovulation and reproductive success. At the moment of release, eggs were sampled for RNA sequencing to compare effects of DHP and P on the expression of genes involved in egg quality aspects. Remaining eggs were fertilized and larval viability was recorded. Both DHP and P were able to induce GVBD (DHP at 10 and 100 ng mL^−1^, P at 100 and 1,000 ng mL^−1^) *in vitro*. Expression of genes involved in oocyte maturation and ovulation was similar *in vitro* for both DHP and P treatments. Regarding the *in vivo* results, RNAseq results reflected similar DHP and P effects on the expression of genes involved in egg quality aspects. Females injected with either DHP or P ovulated, released eggs, and were equally able to produce larvae without any differences in reproductive success. Our results support the conclusion that DHP and P work equally well *in vitro* and *in vivo*. P is more attractive to apply as the price is 3,000 times lower than the price of DHP.

## 1 Introduction

After completing vitellogenesis, the fish oocyte enters the maturation phase which occurs before ovulation and is a prerequisite for successful fertilization. Meiotic maturation (reviewed by Nagahama and Yamashita, 2008) is characterized, among others, by the migration of the germinal vesicle (GV) from the center of the oocyte towards its surface where the nuclear envelope disintegrates (i.e., Germinal Vesicle Breakdown—GVBD). Following maturation, the oocyte escapes from its follicle (i.e., ovulation, reviewed by Takahashi et al., 2019) to be fertilized. Investigations on the mechanisms of oocyte maturation (for review see Nagahama and Yamashita, 2008) and ovulation (for review see Takahashi et al., 2019) have demonstrated that circulatory luteinizing hormone (Lh) levels rise and that Lh binds to its ovarian receptors (Lhcgr) and/or the follicle-stimulating hormone receptor (Fshr) to induce the production of the maturation-inducing hormone (MIH). Once synthetized in the follicular layers, MIH activates the formation and activation in the ooplasm of the maturation-promoting factor (MPF) that induces GVBD (Nagahama and Yamashita, 2008). Following oocyte maturation, MIH binds to its nuclear progestin receptors (Pgrs) (Todo et al., 2000; Hanna et al., 2010) in the granulosa cells to act as a ligand-activated transcription factor (for review see Takahashi et al., 2019). MIH-activated Pgr activates various downstream factors and signaling pathways that are essential for ovulation (Hagiwara et al., 2014; Liu et al., 2017; Takahashi et al., 2019). Among others, Pgr regulates downstream effectors like proteases that are essential for degrading and remodeling the extracellular matrix to allow the release of the mature oocyte (Liu et al., 2017). In fish, MIH also stimulates the production of prostaglandins (PGs) that act through various receptors to trigger the release of the oocyte from its follicles (reviewed by Takahashi et al., 2018). In medaka *Oryzias latipes*, one of the prostaglandin receptors, Ptger4b, seems important for ovulation since its transcript increases drastically before ovulation (Fujimori et al., 2011; Fujimori et al., 2012; Hagiwara et al., 2014).

For inducing sexual maturation, female European eels are weekly injected with pituitary extracts (PE) from carp or salmon to induce oocyte growth. With this protocol, females complete vitellogenesis but often fail to undergo oocyte maturation and ovulation. Therefore, eels are injected with an additional dose of PE followed by 17α,20β-dihydroxy-4-pregnen-3-one (DHP) to induce oocyte maturation and ovulation (Ohta et al., 1996; Adachi et al., 2003; Palstra et al., 2005; Di Biase et al., 2017; Politis et al., 2021). Females injected with DHP must ovulate within 18 h as fertility and hatching rates decreased rapidly after this period in eels (Ohta et al., 1996; Kim et al., 2007; Palstra and van den Thillart, 2009). In the past decades, spawning induction therapies have focused on factors that act higher in the brain-pituitary-gonad axis (bpg axis) to induce the endogenous production of DHP (Mylonas and Zohar, 2007). Progesterone (P, also known as P4), a precursor of DHP in the steroidogenic pathway, has been shown to induce oocyte maturation and ovulation in Japanese eels (Adachi et al., 2003) and other fish species (Zebrafish: Tokumoto et al., 2011; White sturgeon: Webb et al., 2000; Goldeye; Pankhurst, 1985). P is usually considered to indirectly affect oocyte maturation and ovulation due to its conversion into DHP. In salmonids, P, which is formed in the theca cells from pregnenolone, is converted into 17α-hydroxyprogesterone that crosses the basal lamina to be converted into DHP in the granulosa cells (Nagahama, 1997). Assuming a similar mechanism in European eels, P might induce the release of better quality eggs by stimulating the endogenous production of DHP by the eel’s follicles. However, given the capacity of P to effectively induce GVBD at a low dose (10 ng/mL) *in vitro* in *Anguilla japonica* (Adachi et al., 2003), we cannot rule out any direct effect of P on oocyte maturation and ovulation in eels. Therefore, it is possible that upstream precursors in the steroidogenic pathway like P are not converted into DHP but directly affect oocyte maturation.

To our knowledge, the effects of P treatment on oocyte maturation and ovulation have not yet been investigated *in vitro* and *in vivo* in European eels. Therefore, an important question remains to be answered; Is P able to induce oocyte maturation and ovulation *in vitro* and *in vivo* in European eels? P may promote the last steps of oocyte development directly or indirectly by being converted into DHP. To investigate this question, we compared P with DHP and investigated the effects of both treatments on oocyte maturation and ovulation on batch level, *in vitro* and *in vivo*. For that purpose, we developed an *in vitro* culture system of maturing oocytes in the presence of DHP and P to compare their dose-response effects on oocyte maturation and ovulation after 12 and 18 h of incubation. For the *in vivo* study, females were either injected with DHP or P at a dose of 2 mg kg^−1^ and differences in the oocyte transcriptomes were investigated. We hypothesized that P leads to the production of endogenous DHP by the eel’s folliculated oocytes. Alternatively, P may have identical effects as DHP when directly inducing oocyte maturation and ovulation. The consequences of both treatments for egg quality and reproductive success were evaluated.

## 2 Material and methods

### 2.1 Ethics

Both *in vitro* and *in vivo* procedures in European eels were conducted in accordance with the current law of the Netherlands and was approved by the Dutch central committee for animal experimentation (CCD nr. AVD401002017817), the animal experiments committee (DEC) and the animal experimental committee of Wageningen University & Research (IvD nr. 2017. D-0007.004 and 005).

### 2.2 Broodstock conditioning

#### 2.2.1 Males

Both wild and farmed males of 80–120 g were used for the *in vivo* experiment. Wild eels were caught in the Harinxma canal (Netherlands). Farmed eels were obtained from the eel farm Palingkwekerij Koolen B.V (Bergeijk, Netherlands). Both wild and farmed males were transported to the animal experimental facilities (CARUS, Wageningen, Netherlands) and transferred to tanks connected to a recirculating system with artificial seawater (16°C, 36 ppt).

#### 2.2.2 Females

For the *in vitro* trials, feminized eels were used. For the *in vivo* trials, feminized eels and wild females were used to compare the effects of feminization on treatment.

Elvers of 10 g were transferred from the eel farm Palingkwekerij Koolen B.V. (Bergeijk, Netherlands) to the animal experimental facilities of Wageningen University & Research (CARUS, Netherlands). After arrival, they were placed in 400-L tanks kept in freshwater (FW) and 24°C under dimmed light conditions. For inducing feminization, elvers were fed with 17β-estradiol (E2) coated pellets for 6 months (Chai et al., 2010). Following the feminization procedure, females were fed a broodstock diet for an additional 6 months. Premature females of ∼400 g were then selected, transferred to a 3,600-L swim-gutter with seawater (Tropic Marine, 36 ppt) and subjected to simulated migration (slightly adjusted protocol from Mes et al., 2016) for 2 months to initiate sexual maturation. The simulated migration consisted of constant swimming against a flow with a speed of 0.5 m s^−1^, in the dark at daily alternating temperatures between 10°C and 15°C. After the simulated migration, females were anesthetized with 2-phenoxyethanol (2 mL in 10 L water), PIT tagged (TROVAN, Aalten, Netherlands) and transferred to 373-L tanks connected to a recirculating system with artificial seawater (16°C, 36 ppt). Each female was injected in the peritoneal activity with a steroid implant containing 17-methyltestosterone (5 mg) and E2 (2 mg) for 2 months to induce the start of vitellogenesis (Palstra et al., 2022).

Wild silver eels of ∼500g were caught during their seaward migration in the Van Harinxma canal (Friesland, Netherlands). Females were transported to the animal experimental facilities (CARUS, Wageningen, Netherlands). After arrival, females were anesthetized, PIT tagged (TROVAN, Aalten, Netherlands), acclimatized in tanks (16°C, 36 ppt) for 13 weeks and injected with hCG at a dose of 3000 IU.kg^−1^ (Chorulon, MSD, Kenilworth, New Jersey, United Stated). One month later, wild females were injected in the peritoneal cavity with a steroid implant containing E2 (2 mg) for 2 months to induce the start of vitellogenesis (Palstra et al., 2022).

### 2.3 *In vitro* experiment

#### 2.3.1 Artificial maturation

Eleven feminized females were artificially matured according to the previously described routine protocol (Palstra et al., 2005). Feminized females were weekly intramuscular injected with 20 mg kg^−1^ CPE to induce further vitellogenic growth (Catfish, Den Bosch, Netherlands). Starting in week 7, 2 days after each injection, females were weighed to determine the body weight index (BWI: body weight/body weight at the moment of first CPE injection × 100%) and body girth index (BGI: body girth/body length). When BWI > 110% and BGI > 0.24, females were anesthetized and sampled for ovarian tissue by inserting a cannula through the cloaca. Oocyte development was then graded on a scale from 1 to 7 according to Palstra et al. (2005). When oocytes were on average in stage 3 (i.e., transparent oocytes with migrating germinal vesicle), females were given an additional CPE injection at a dose of 20 mg kg^−1^ to booster oocyte maturation. One day later, females were checked again to assess the progression of oocyte maturation. When oocytes developed to stage 4 (i.e., transparent oocytes with germinal vesicle at the periphery), females are commonly injected with DHP at 2 mg kg^−1^ to induce oocyte maturation and ovulation (Ohta et al., 1996; Palstra et al., 2005). Therefore, ovarian tissues (∼5 g) were taken by needle (inner diameter of 2.3 mm) from a standardized location (5 cm anterior from the cloaca) and used for *in vitro* incubation studies.

#### 2.3.2 Chemicals

DHP (Cayman, Michigan, United States) and P (Sigma-Aldrich, Saint Louis, MO, United States) powders were initially dissolved in absolute ethanol. Stock solution of DHP and P were further dissolved in Gibco™ Leibovitz’s L15 medium GlutaMAX supplement (Thermo Fisher Scientific, Waltham, MA, United States) and aliquots were made of the desired concentration. In the aliquots, ethanol concentration was below 0.1%.

#### 2.3.3 *In vitro* dose-response effects of ovarian tissue

Immediately after extraction, ovarian tissues were placed in ice-cold culture medium supplemented with 2.5 g L^−1^ HEPES, 0.1 g L^−1^ streptomycin and 100,000 IU L^-1^ penicillin (Pen Strep, ThermoFisher, Waltham, MA, United States). For one female, twenty oocytes were placed in 4% buffered paraformaldehyde, refrigerated at 5°C overnight and stored in 70% ethanol for later confirmation of the integrity of the oocyte follicles. For each female, 60 oocytes were randomly stocked per well in 21 wells of two 24-well plates. In each plate, oocytes were stocked in 1 mL medium supplemented with the treatment (DHP at 1, 10 and 100 ng mL^−1^ or P at 10, 100 and 1,000 ng mL^−1^) or with hormone free media for the control group, and tested in triplicate. The difference in applied concentrations of DHP and P is based on the higher potency of DHP vs. P to activate the receptor (Todo et al., 2000). One culture plate was incubated for 12 h and one plate was incubated for 18 h at 20°C to mimic the *in vivo* conditions. Just before incubating (at 0 h), the remaining oocytes were sampled for microscopy and gene expression analysis as described in the following two sections. After incubations, oocytes were again sampled for microscopy and gene expression analysis.

#### 2.3.4 Microscopy analysis

For each female, a total number of 20 oocytes that represented stage 2 (i.e., transparent oocyte at the start of GV migration) and further, were used for microscopy analysis according to the oocyte scale developed by Palstra et al. (2005). Oocytes were placed in Serra’s fix (ethanol:formalin:acetic at 6:4:1, diluted ×20 in PBS) for 3 min to stain the GV. When maturing oocytes lacked the GV, GVBD was considered to have occurred. Stained oocytes were photographed for later assessment of the percentage of oocytes that displayed GVBD and measurements of the lipid droplet and oocyte diameter. Lipid droplet diameters were measured according to Unuma et al. (2011) with the open source software ImageJ. On visual assessment, the ten largest lipid droplets were measured and the five maximum values averaged for each oocyte. For determination of the oocyte diameter, the maximum diameter was measured.

#### 2.3.5 Histology

Oocytes were fixed in cold 4% buffered paraformaldehyde, kept refrigerated at 5°C overnight and then preserved in 70% ethanol. Ten oocytes were randomly selected, placed in 0.9% type II low gelling agarose (Sigma Aldrich, Saint Louis, MO, United States), dehydrated via an ethanol/xylene series and embedded in paraffin. Oocytes were then sectioned using a microtome into 5 µm thick sections and stained with Mayer’s hematoxylin & eosin. Oocytes were photographed with a Leica DFC450c color camera attached to a Leica DM6b microscope.

#### 2.3.6 Gene expression analysis

For each female, a total number of 20 oocytes including different stages from stage 3 onwards were selected under the binocular according to the oocyte scale developed by Palstra et al. (2005). Stage 0 (i.e., opaque oocytes), stage 1 (i.e., opaque oocytes with a centered nucleus becoming visible) and stage 2 (i.e., fully transparent oocyte containing numerous oil droplets) were excluded from the gene expression analysis since these oocytes are unlikely to respond to DHP and P. After selection, oocytes were photographed, placed in 1 mL RNAlater (Ambion Inc., Huntingdon, United Kingdom), kept refrigerated at 5°C overnight and then stored at −80°C until RNA extraction.

For RNA extraction, oocytes were homogenized with a tissue lyzer (Qiagen, Tissuelizer II) in 1 mL Trizol (Invitrogen, California, United States). Possible contaminant traces of DNA were digested with ISOLATE II RNA Mini Kit (Bioline, London, United Kingdom). After RNA extraction and DNase treatment, RNA purity was assessed by the 260/280 ratios (2.2 ± 0.1) on the Nanodrop (ThermoFisher, Waltham, MA, United States) and, by the absence of RNA breakdowns, on the 2100 Bioanalyzer (Agilent, Santa Clara, CA, United States). Complementary DNA was generated from RNA using random primers and dNTPs with Superscript III Reverse transcriptase (ThermoFisher, Waltham, MA, United States).

Gene expression analysis was performed as previously described (Jéhannet et al., 2021). Diluted complementary DNA was mixed with SensiFAST™ SYBR^®^ Lo-ROX Ki (Bioline, London, United Kingdom) and the primer set of the target gene (Table 1). Since the gene expression analysis data could not be obtained from a single 96-well plate due to the high amount of samples, several plates were run per gene. For comparing the data obtained from the different plates, the following were taken into consideration 1) reference and target genes were run on the same 96-well plate; 2) the same master mix (SensiFAST SYBR and primer set) was used between plates and 3) samples were randomized through the plates. The conditions in the QuantStudio Real-time PCR system (ThermoFisher, Waltham, MA, United States) used were 95°C during 2 min followed by 40 cycles of denaturation at 95°C for 5 s, annealing at 60°C–64°C for 10 s and extension at 72°C for 5 s. Primer-dimers artifacts and reaction specificity were checked by melt curve analysis. PCR products were electrophoresed on 1.5% agarose gel. Standard curves that were made by diluting sample cDNA had R^2^ values above 0.98 and efficiencies between 92% and 109%. For the very few samples that did not amplify due to low gene expression, CT values were manually set at 35. Transcripts levels of each target were normalized over *elf1* (no difference in expression between treatments; ANOVA, *p* = 0.341) and data were expressed as fold change by using the 2^−ΔΔCT^ method from Livak and Schmittgen (2001).

**TABLE 1:** Primers used for each target gene with T⁰: annealing temperature and bp: base pair.

Gene	Accession number	Primer sequence	T⁰	Length	Efficiency (%)	Source
*elf1*	EU407825	FW: CCC​CTG​CAG​GAT​GTC​TAC​AA	64	152 bp	96	Setiawan and Lokman (2010)
		RV: AGG​GAC​TCA​TGG​TGC​ATT​TC				
*pgr1*	AFV13730.1	FW: AGT​TTG​CCA​ATC​TCC​AGG​TG	60	107 bp	101	Morini et al. (2017)
		RV: ATC​AAA​CTG​TGG​CTG​GCT​CT				
*pgr2*	AFV13731.1	FW: GCC​TCT​GGA​TGT​CAC​TAC​GG	60	95 bp	94	Morini et al. (2017)
		RV: CCG​GCA​CAA​AGG​TAG​TTC​TG				
*fshr*	LN831181	FW: CCT​GGT​CGA​GAT​AAC​AAT​CAC​C	63	173 bp	109	Zadmajid et al. (2015)
		RV: CCT​GAA​GGT​CAA​ACA​GAA​AGT​CC				
*lhcgr1*	LN831182	FW: GCG​GAA​ACA​CAG​GGA​GAA​C	60	155 bp	101	Maugars and Dufour (2015)
		RV: GGT​TGA​GGT​ACT​GGA​AAT​CGA​AG				
*lhcgr2*	LN831183	FW: TCA​ACA​ACC​TCA​CCA​ATC​TCT​CT	62	162 bp	106	This study
		RV: GCA​GTG​AAG​AAA​TAG​CCG​ACA				
*ptger4b*	XM_035392436.1	FW: ATT​GAG​AAG​GTG​AAG​TGC​CTG​T	62	169 bp	105	This study
		RV: AGA​ATG​TTT​GAG​AGG​TGC​TGG​T				
*arα*	FR668031	FW: AGG​AAG​AAC​TGC​CCC​TCT​TG	62	90 bp	93	Setiawan and Lokman (2010)
		RV: ATT​TGC​CCG​ATC​TTC​TTC​AG				

### 2.4 *In vivo* experiment

#### 2.4.1 Artificial maturation

Twenty-two feminized and eighteen wild females were artificially matured according to the procedure described in Section 2.2.2. For ovulation induction, females were either injected with DHP or P at a dose of 2 mg kg^−1^ (Ohta et al., 1996) Just before the DHP or P injection, ovarian tissues were extracted from feminized eels with a cannula. Twenty oocytes were selected (see Section 2.3.6) and stored in RNAlater (Ambion Inc., Huntingdon, United Kingdom) for RNAseq analysis. Females were then placed in tanks and water temperature was gradually increased from 18 to 20°C; the optimum temperature during induction of spawning in eels (Unuma et al., 2012). In the timespan of 11–18 h after receiving the injection, females were regularly checked for egg release. When eggs could be retrieved, females were stripped by applying gentle pressure on the abdomen. At the moment of stripping, eggs from feminized females were sampled again for RNAseq analyses to compare treatment effects of DHP and P on stripped eggs.

Males were injected with 1,000 IU human Chorionic Gonadotropin (hCG: Kahn et al., 1987) and placed back in the 373-L tanks kept at 36 ppt and 16°C. One day before fertilization, males were anesthetized and checked for spermiation by applying gentle pressure on the abdomen. Males that were spermiating were injected with 250 IU hCG, dissolved in 0.9% physiological salt solution, to increase sperm quality. Males were placed in the spawning tank with the DHP or P injected females. When females were ready to be stripped, males (*N* = 6–10) were anesthetized and 2–3 mL of sperm was taken which was directly diluted into 45 mL artificial eel plasma (Peñaranda et al., 2010).

#### 2.4.2 Fertilization and egg incubation

After collection of the stripped eggs into plastic bowls, the diluted sperm was dripped onto the eggs. Gametes were mixed by stirring. Artificial seawater (36 ppt, 18°C) was added for a contact time of 5 min. After 5 min, 10 g of eggs were placed in a 100 mL cylinder filled with artificial seawater to determine the percentage of floating eggs in relation to the eggs that were sinking 1 hour later. The remaining eggs were placed in 3L-beakers filled with artificial seawater. After 1 h, the floating egg layer was collected from the 3L-beakers, rinsed gently over a sieve and transferred into beakers filled with fresh artificial seawater. Eggs were kept into suspension by gentle aeration and dead material was removed every 12 h to prevent microbial infections. After hatching (48–60 h post fertilization), the content of each beaker was gently mixed to uniformly disperse the larvae in the water column. Then, a water sample of 20 mL was taken to count the number of larvae and extrapolate this number per volume to the 3L-beakers. Larvae were gently transferred to plankton nets that were hanging in conic tanks filled with seawater and connected to a 338 L recirculating system. Larval survival was daily recorded until all larvae of the particular batch had died.

#### 2.4.3 RNA sequencing

RNA-seq was performed on oocytes and eggs from three feminized females for each treatment (DHP, P). RNA was extracted using the miRNeasy Mini Kit (Qiagen, Venlo, Netherlands). Integrity and concentration of the RNA samples were determined using an Agilent TapeStation 4200 device. RNA concentrations varied from 132 to 796 ng μL^−1^ and RIN values were between 7.0 and 10.0. Barcoded RNA-seq libraries were prepared from 0.5 μg of total RNA using the TruSeq Stranded mRNA Library Prep kit according to the manufacturer’s instructions (Illumina Inc., San Diego, CA, United States). RNA-seq libraries were sequenced on an Illumina NovaSeq 6000 System according to the manufacturer’s protocol. Image analysis and base calling were done by the Illumina pipeline (Illumina Inc., San Diego, CA, United States). RNA-seq data yield varied from ∼12 to ∼16 million paired-end 2 × 150 bp reads per sample, corresponding to ∼3.7–4.9 Gb per sample.

Quantitative analysis of the RNA-seq datasets was performed by alignment of reads against the *Anguilla anguilla* reference genome (https://www.ncbi.nlm.nih.gov/data-hub/genome/GCF_013347855.1/) using TopHat (version 2.0.13) (Trapnell et al., 2009). In total between ∼54% and ∼67% of the RNA-Seq reads could be mapped against the reference. The resulting files were filtered and secondary alignments were removed using SAMtools (version 1.2 using htslib 1.2.1) (Li et al., 2009). For comparison of gene expression levels between groups, aligned fragments per predicted gene were counted from SAM alignment files using the Python package HTSeq (version 0.6.1p1) (Anders et al., 2014). In order to make comparisons across samples possible, these fragment counts were corrected for the total amount of sequencing performed for each sample. As a correction scaling factor, we employed library size estimates determined using the R/Bioconductor package DESeq (Anders and Huber, 2010). Read counts were normalized by dividing the raw counts obtained from HTSeq by its scale factor. Aligned reads were processed using DESeq whereby treatment groups were each compared with the control group. Raw and processed RNA-Seq datasets have been submitted to NCBI’s GEO repository with reference GSE218444 (https://www.ncbi.nlm.nih.gov/geo/query/acc.cgi?acc=GSE218444; GSM6745397-GSM6745408). The comparisons between 1) oocytes sampled before the DHP injection and eggs stripped after DHP and 2) oocytes sampled before the P injection and eggs stripped after P were analyzed to assess differential gene expression and their functional clustering by GO analysis using UniProt (https://uniprot.org). Differences were considered significant when *p* < 0.01.

### 2.5 Statistical analysis

#### 2.5.1 In vitro

Fold change of each target gene, oocyte diameter, lipid diameter and the percentage of GVBD were compared between timepoints and doses of DHP by using the following linear mixed model:

$$\ln\left(y_{ijkl}\right)=\mu +{TR}_{i}+T_{j}+{TR}_{i}\times T_{j}+A_{k}+P_{l}+e_{ijkl}$$

Where $y_{ijkl}$ is the variable to be explained (fold change of each target gene, oocyte diameter, lipid diameter and GVBD) from treatment i (control, 1 ng DHP, 10 ng DHP, 100 ng DHP) at incubation time j (0, 12 and 18 h) observed in eel k (k = 1–11) and kept in plate l (l = 1–22). In this model, μ is the overall mean, ${TR}_{i}$ is the fixed effect of the i ^th^ treatment where *i* is the hormonal concentration, $T_{j}$ is the fixed effect of the j ^th^ time, ${TR}_{i}\times T_{j}$ is the fixed interaction effect for the i ^th^ treatment and j ^th^ time, $A_{k}$ is the random effect of the k ^th^ eel, $P_{l}$ is the random effect of the l ^th^ plate, and $e_{ijkl}$ is the residual. The same linear mixed model was used to investigate the effect of P treatment on the fold change of each target gene, oocyte diameter, lipid diameter and GVBD. All random effects were assumed to be independent and normally distributed. Data were analyzed using R (R foundation for statistical computing, Vienna, Austria) and considered significant when *p* < 0.05.

#### 2.5.2 In vivo

The number of injections to reach maturation, the timespan after the DHP or P injection until egg release, the percentage of floating eggs, the number of larvae after hatching and larval survival were compared between DHP and P injected females with the non-parametrical Wilcoxon rank sum test. Data were analyzed with R and differences were considered significant when *p* < 0.05.

## 3 Results

### 3.1 *In vitro* experiment

#### 3.1.1 Histology

**FIGURE 1:**
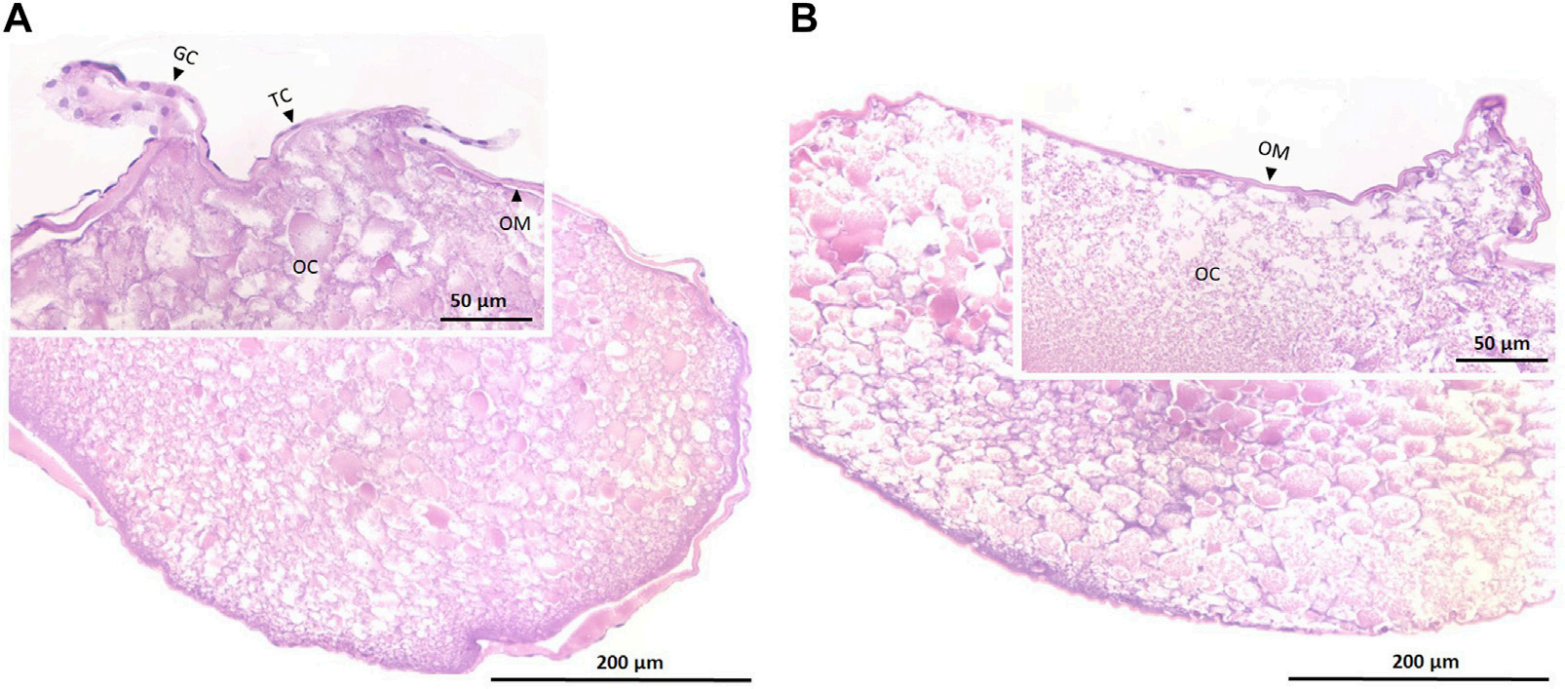
Histological sections of maturing oocytes in European eel that are folliculated **(A)** and de-folliculated **(B)**. Insets show parts of sections at higher magnification. OC, oocyte; OM, oocyte membrane; TC, theca cells and GC; granulosa cells. Contrary to the de-folliculated oocytes, folliculated oocytes still show some theca and granulosa cells (Tosaka et al., 2010).

Oocyte samples that were used for the *in vitro* experiment were a mix of both folliculated and de-folliculated oocytes (Figure 1). While some oocytes were enveloped by theca and granulosa cells (Figure 1A), other oocytes were only surrounded by their membranes (Figure 1B).

#### 3.1.2 GVBD, hydration and lipid fusion

**FIGURE 2:**
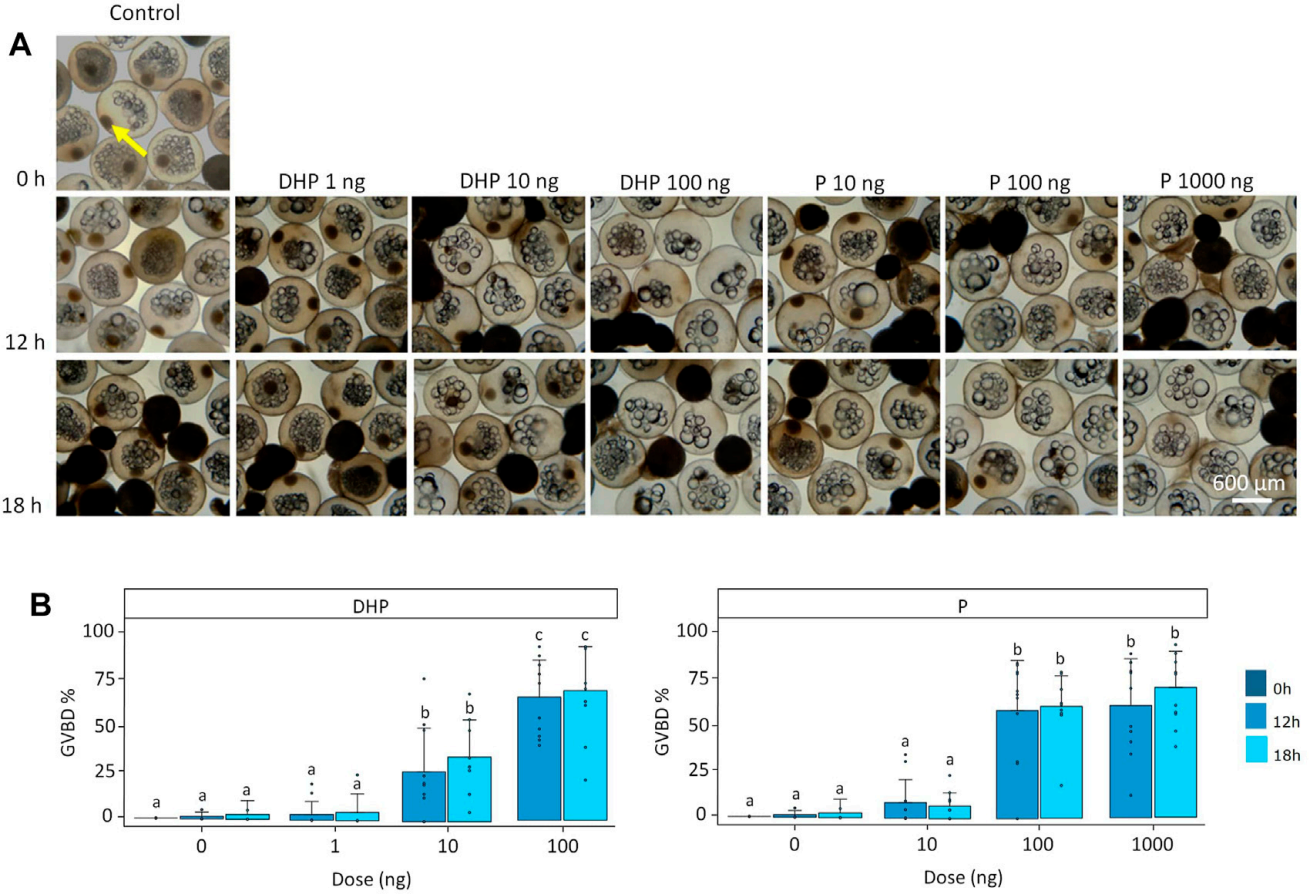
Maturing oocytes and germinal vesicle breakdown (GVBD). **(A)** Maturing oocytes fixed in Serra’s solution to stain the germinal vesicle GV (yellow arrow) and **(B)** percentage of oocytes where GVBD had occurred shown as percentage (GVBD%). Oocytes were treated with various doses of DHP (0, 1, 10, and 100 ng mL^−1^) and P (0, 10, 100, and 1,000 ng mL^−1^) *in vitro* at the start of incubation (dark blue; hardly visible as GVBD% was 0), and after 12 (mild blue) and 18 h (light blue) of incubation. Oocytes incubated without hormone have a visible GV at the start and after 12 and 18 h of incubation. Similarly, oocytes incubated at low doses of DHP (1 ng) and P (10 ng) still have a visible GV after 12 and 18 h of incubation. Some oocytes incubated with doses of 10 ng DHP after 12 and 18 h of incubation did not show presence of a GV since GVBD was induced in ∼27% of oocytes. Most oocytes incubated with doses of 100 ng DHP, 100 ng P and 1,000 ng P after 12 and 18 h of incubation did not show presence of a GV since GVBD was induced in ∼70% of oocytes. GVBD% was compared between timepoints and doses for DHP and P. Bars with no overlap in letters are significantly different (*p* < 0.05). Data are displayed as barplots with averages ± standard deviation and individual datapoints as circles. Data are based on oocytes originating from *N* = 11 eels.

Both DHP and P treatments (*p* < 0.001), but not time (*p* > 0.05), significantly affected GVBD (Figures 2A, B). Also, a significant eel effect (*p* < 0.05) was detected. Oocytes incubated at a dose of 100 ng DHP underwent GVBD at a higher rate than oocytes treated at a dose of 10 ng DHP (Figure 2B). After 12 h of incubation, GVBD was observed in 27% of the oocytes at doses of 10 ng DHP and in 67% of the oocytes at 100 ng DHP (Figure 2B). After 18 h of incubation, GVBD was observed in 35% of the oocytes at doses of 10 ng DHP and in 71% of the oocytes at 100 ng DHP (Figure 2B). When incubating ovarian tissues with P, maturing oocytes were lacking a GV at doses of 100 ng P and 1,000 ng P (Figure 2B). After 12 h of incubation, GVBD was observed in 63% of the oocytes at doses of 100 ng P and in 65% of the oocytes at 1,000 ng P (Figure 2B). After 18 h of incubation, GVBD was observed in 65% of the oocytes at doses of 100 ng P and in 75% of the oocytes at 1,000 ng P (Figure 2B). No statistical differences were detected between 100 ng P and 1,000 ng P (Figure 2B).

**FIGURE 3:**
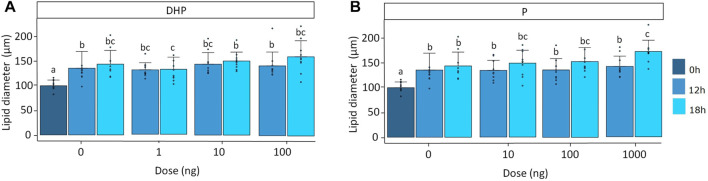
Lipid diameter when treated with various doses of **(A)** DHP (0, 1, 10, and 100 ng mL^−1^) and **(B)** P (0, 10, 100, and 1,000 ng mL^−1^) at the start of incubation (dark blue), and after 12 (mild blue) and 18 (light blue) hours of incubation *in vitro*. Measurements were compared between timepoints and doses for DHP and P. Lipid diameter increased over time as a result of fusion of lipid droplets. Treatment with a dose of 100 ng DHP after 18 h of incubation tended to increase lipid diameter but results were not statistically different from the 18 h control; probably due to the high variations in the data. Treatment with a dose of 1,000 ng P after 18 h of incubation induced lipid fusion. Bars with no overlap in letters are significantly different (*p* < 0.05). Data are displayed as barplots with averages ± standard deviation and individual datapoints as circles. Data are based on oocytes originating from *N* = 10 eels.

For both DHP and P, lipid diameter was significantly affected by time (*p* < 0.001) and treatment (*p* < 0.05). Moreover, a significant eel (*p* < 0.001) effect was observed. Lipid diameter increased from 101 ± 18 μm at the start of the incubation to 137 ± 34 µm after 12 h of incubation and 146 ± 38 µm after 18 h of incubation in the controls (Figure 3). At a dose of 100 ng DHP after 18 h of incubation, the lipid diameter increased from 146 ± 38 μm to 163 ± 33 µm which was not statistically different from the 18 h control, probably due to the high variation in the data (Figure 3A). At a dose of 1,000 ng P after 18 h of incubation, the lipid diameter significantly increased from 146 ± 38 μm to 176 ± 23 µm when compared to the 18 h control (Figure 3B). Oocyte diameter did not change with time nor treatment (Supplementary Figure S1).

#### 3.1.3 Expression of nuclear progestin receptors

**FIGURE 4:**
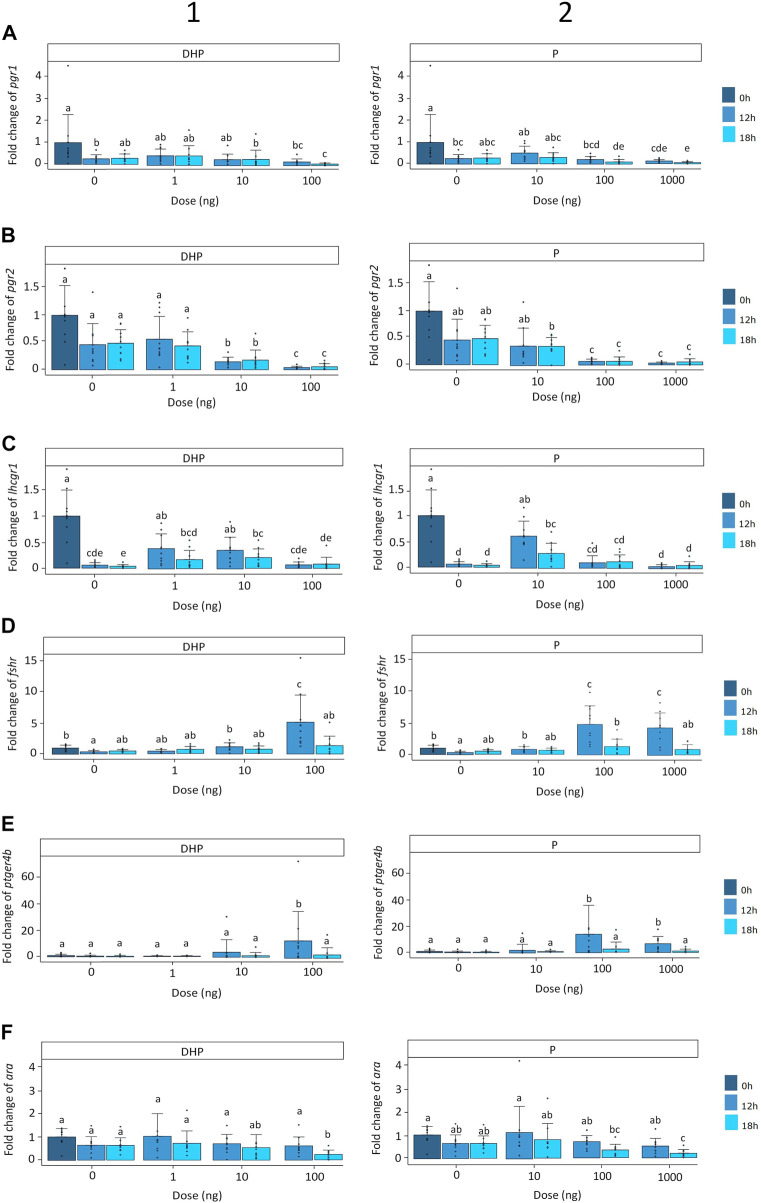
Expression of the **(A)** nuclear progestin receptors *pgr1*, **(B)** nuclear progestin receptors *pgr2*, **(C)** luteinizing hormone receptor *lhcgr1,* **(D)** follicle-stimulating hormone receptor *fshr*, **(E)** prostaglandin receptor *ptgr4b* and **(F)** androgen receptor *ara* when treated with various doses of (**1**) DHP (0, 1, 10, and 100 ng mL^−1^) and (**2**) P (0, 10, 100, and 1,000 ng mL^−1^) at the start of incubation (dark blue) and after 12 (mild blue) and 18 (light blue) hours of incubation *in vitro*. Receptor expression was normalized to the start of incubation and expressed as fold change. Expression was compared between timepoints and doses for DHP and P. Expression of *pgr1* was decreased in oocytes incubated with doses of 100 ng DHP, 100 ng P and 1,000 ng P after 18 h of incubation. Expression of *pgr2* was decreased in oocytes incubated with doses of 10 ng DHP, 100 ng DHP, 100 ng P and 1,000 ng P after 12 and 18 h of incubation. Expression of *lhcgr1* decreased with time and treatment. The lowest doses of DHP (1 and 10 ng) increased the expression of *lhcgr1* after 12 and 18 h of incubation when compared to the corresponding controls. Oocytes incubated with the lowest dose of P (10 ng) followed a similar expression pattern than oocytes incubated with DHP. Expression of *fshr* was increased in oocytes incubated with doses of 100 ng DHP, 100 ng P and 1,000 ng P after 12 h of incubation. Similarly, doses of 100 ng DHP, 100 ng P and 1,000 ng P increased the expression of *ptger4b* after 12 h of incubation. Expression of *ara* was decreased with doses of 100 ng DHP and 1,000 ng P after 18 h of incubation. Bars with no overlap in letters are significantly different (*p* < 0.05). Data are displayed as barplots with averages ± standard deviation and individual datapoints as circles. Data are based on oocytes originating from *N* = 10 eels.

For both DHP and P, expression of the nuclear progestin receptor *pgr1* was significantly affected by time (*p* < 0.01), treatment (*p* < 0.001) and time × treatment interaction (*p* < 0.05), and a significant eel effect (*p* < 0.001) was observed. *Pgr1* expression decreased over 4-fold with time and over 15-fold after 18 h of incubation, for both treatments (Figure 4A). At a dose of 100 ng DHP, *pgr1* expression decreased after 18 h of incubation when compared to the controls (Figure 4A1). Similarly, at the highest doses of P (100, 1,000 ng), *pgr1* expression decreased after 18 h of incubation when compared to the controls (Figure 4A2). No statistical differences were detected between the doses of 100 ng and 1,000 ng P after 18 h of incubation (Figure 4A2).

For both DHP and P, expression of the nuclear progestin receptor *pgr2* was significantly affected by time (*p* < 0.05) and treatment (*p* < 0.001), and a significant eel effect (*p* < 0.01) was observed. Expression of *pgr2* decreased over 2-fold with time and over 20-fold after 12 and 18 h of incubation for both treatments (Figure 4B). At doses of 10 ng DHP, 100 ng DHP, 100 ng P and 1,000 ng P, the expression of *pgr2* decreased after 12 and 18 h of incubation when compared to the controls (Figure 4B). A dose of 100 ng DHP decreased the expression of *pgr2* further than a dose of 10 ng DHP (Figure 4B1). No statistical differences were detected between doses of 100 ng and 1,000 ng P (Figure 4B2).

#### 3.1.4 Expression of the luteinizing hormone receptors

For both DHP and P, expression of the luteinizing hormone receptor *lhcgr1* was significantly affected by time (*p* < 0.001) and treatment (*p* < 0.05) and a significant eel effect (*p* < 0.05) was observed. Expression of *lhcgr1* decreased over 20-fold with time and remained low with exception of the lower doses for both treatments after 12 and 18 h of incubation (Figure 4C). When incubating ovarian tissues with DHP for 12 and 18 h, at the lower doses of DHP (1 and 10 ng), but not DHP at 100 ng, the expression of *lhcgr1* increased when compared to the controls (Figure 4C1). Similarly, at the lowest dose of P (10 ng) after 12 and 18 h of incubation, but not the highest ones (P 100 ng, P 1,000 ng), the expression of *lhcgr1* increased when compared to the controls (Figure 4C2). The expression of the luteinizing hormone receptor *lhcgr2* remained low and stable with time and treatment; significant differences were detected but differences were not correlated with time and treatment (Supplementary Figure S2).

#### 3.1.5 Expression of follicle stimulating hormone receptor and prostaglandin receptor

For both DHP and P, expression of the follicle stimulating hormone receptor *fshr* was significantly affected by time (*p* < 0.05) and time × treatment interaction (*p* < 0.05) and a significant eel (*p* < 0.01) effect was detected. *Fshr* expression remained low and stable with exception of a 5-fold increase after 12 h of incubation for both treatments (Figure 4D). When ovarian tissues were incubated with DHP, treatment did not affect *fshr* expression (*p* > 0.05) with exception of one dose after 12 h of incubation. A dose of 100 ng DHP significantly increased *fshr* expression after 12 h of incubation when compared to the controls and the lowest DHP concentrations (1, 10 ng) (Figure 4D1). For P, treatment significantly affected *fshr* expression (*p* < 0.05). Doses of 100 ng and 1,000 ng P increased the expression of *fshr* after 12 h of incubation when compared to the controls and lowest P concentration (10 ng) (Figure 4D2). No statistical differences were detected between the doses of 100 and 1,000 ng P after 12 h of incubation (Figure 4D2).

For both DHP and P, expression of the prostaglandin receptor *ptger4b* was significantly affected by time × treatment interaction (*p* < 0.001) and a significant eel (*p* < 0.01) effect was detected. *Ptger4b* expression remained low and stable with exception of a 13-fold increase after 12 h of incubation for both treatments (Figure 4E). When ovarian tissues were incubated with DHP, treatment did not affect *ptger4b* expression (*p* > 0.05) with exception of 100 ng DHP which increased the expression of *ptger4b* after 12 h of incubation when compared to the controls and doses of 1 ng DHP and 10 ng DHP (Figure 4E1). P treatment significantly affected *ptger4b* expression (*p* < 0.05). Ovarian tissues incubated with P increased *ptger4b* expression in a similar manner as DHP treatment (Figure 4E2). At doses of 100 ng and 1,000 ng P, the expression of *ptger4b* increased after 12 h of incubation when compared to the controls and dose of 10 ng P (Figure 4E2). No statistical differences were detected between 100 and 1,000 ng P after 12 h of incubation (Figure 4E2).

#### 3.1.6 Expression of androgen receptor

For both DHP and P, treatment (*p* < 0.05) and time × treatment interaction (*p* < 0.05) significantly affected the androgen receptor *ara* expression. A significant eel effect (*p* < 0.05) was detected. *Ara* expression remained low and stable with exception of a 4-fold decrease after 18 h of incubation for both treatments (Figure 4F). When incubating ovarian tissues with DHP, at a dose of 100 ng DHP, *ara* expression decreased after 18 h of incubation when compared to the controls (Figure 4F1). Similarly, at the highest dose of P (1,000 ng), the expression of *ara* decreased after 18 h of incubation when compared to the controls (Figure 4F2). No statistical differences were detected between doses of 100 and 1,000 ng P after 18 h of incubation (Figure 4F2).

### 3.2 *In vivo* experiment

#### 3.2.1 Reproductive success in feminized and wild eels

An overview of the reproductive success of feminized females injected with DHP or P is shown in Table 2. The number of weekly CPE injections to reach maturation was similar between females injected with DHP (8.2 ± 1.2 injections) and P (8.0 ± 1.4 injections) (*p* = 0.51). Also the timespan between treatment and egg release was similar between DHP (13.8 ± 1.7 h) and P (14.0 ± 0.7 h) injected females (*p* = 0.42). Of the twelve eels that were injected with DHP, five died, one was stripped but did not give embryos and six eels gave larvae that survived until 8 dph. Of the ten eels that were injected with P, five died, one was stripped but did not give embryos and four eels gave larvae that survived until 5 dph. The percentage of floating eggs was similar between DHP (average: 36%) and P (average: 27%) injected females (*p* = 0.87). Expected differences in larvae number and survival between the DHP and P injected females were not significant (DHP: 6 females; P: 4 females).

**TABLE 2:** Reproductive success of 22 feminized European eels after 17α,20β-dihydroxy-4-pregnen-3-one (DHP) or progesterone (P) injection.

Tag	Injection	Nb.	BWI_1_	BWI_2_	BGI_1_	BGI_2_	t(h)	Floating eggs (%)	Larvae nr.	Survival	Fate
11 EC	DHP	8		125		0.25	13.25	20	50	6	Gave larvae
DC73	DHP	9	122	133	0.26	0.27	14.25	95	100	8	Gave larvae
2653	DHP	7	117	124	0.26	0.27	12	50	200	6	Gave larvae
3745	DHP	8	114	124	0.24	0.27	12.75	50	2,000	5	Gave larvae
4C37	DHP	7	113	121	0.25	0.27	12.25	20	100	4	Gave larvae
BEFA	DHP	7		114		0.25	16	15	50	6	Gave larvae
DDCC	DHP	7	113	124	0.24	0.26	16	1			No embryos
0CEB	DHP	7	118	124	0.22	0.24					Died after DHP
DD56	DHP	7	120	125	0.26	0.26					Died after DHP
D3EA	DHP	7	121	126	0.24	0.25					Died after DHP
6647	DHP	7	106	111	0.24	0.24					Died after DHP
D8F0	DHP	11	125	127	0.26	0.26					Died after DHP
E13F	P	10	120	138	0.23	0.26	15	30	10	1	Gave larvae
1FB8	P	9	114	124	0.25	0.26	15.5	30	10	5	Gave larvae
34AD	P	8	117	119	0.25	0.27	13.75	30	50	3	Gave larvae
CA3C	P	7		121		0.27	14	45	10	4	Gave larvae
E676	P	8	113	120	0.24	0.27	14.75	0			No embryos
2B08	P	7	126	133	0.27	0.29					Died after P
42AD	P	11	114	116	0.26	0.26					Died after P
D8E1	P	7		129		0.27					Died after P
DE5C	P	8		104		0.25					Died after P
D28B	P	7	108	110	0.25	0.25					Died after P

Tag: code of the female, Injection: type of injection that the female received; Nb: number of weekly injections to reach maturation, BWI_1_: body weight index (BWI) at booster moment, BWI_2_: BWI at DHP/P injection, BGI_1_: body girth index (BGI) at booster moment, BGI_2_: BGI at DHP/P injection, t(h): hours after DHP/P injection, Floaters(%): percentage of floating eggs 1 h after fertilization, Larvae nr.: number of larvae that hatched; Survival: number of days that the larvae survived after hatching.

An overview of the reproductive success of wild females injected with DHP and P is shown in Table 3. The number of weekly CPE injections to reach maturation was similar for the eels used in both treatment groups. Eels that required 9 ± 1.5 CPE injections were injected with DHP, eels that required 9 ± 1.0 CPE injections were injected with P (*p* = 0.26). The timespan between treatment and egg release was similar between DHP (11.9 ± 1.1 h) and P (12.5 ± 2.3 h) injected females (*p* = 0.75). When comparing wild and feminized eels, wild females released eggs sooner after treatment (12.2 ± 1.7 h) than the feminized ones (14.1 ± 1.4 h) (*p* = 0.004). Of the nine females that were injected with DHP, two died and seven eels were stripped and gave larvae that survived until 20 dph. Of the nine wild females that were injected with P, two died, one released eggs but did not give embryos and six were stripped and gave larvae that survived until 21 dph. No significant differences were detected between the DHP and P injected females regarding reproductive success. The percentage of floating eggs ranged from 18% to 92% for the eels that received DHP treatment (average 54%) and from 1% to 92% for eels that received P treatment (average: 63%). The number of larvae ranged from 50 to 20,000 for DHP treatment (average: 2,993 larvae) and from 20 to 45,000 for P treatment (average: 12,753 larvae). Larvae survived on average for 7 dph for each of the two treatments.

**TABLE 3:** Reproductive success of 18 wild females European eels after 17α,20β-dihydroxy-4-pregnen-3-one (DHP) or progesterone (P) injection.

Tag	Injection	Nb.	BWI_1_	BWI_2_	BGI_1_	BGI_2_	t(h)	Floating eggs (%)	Larvae nr.	Survival	Fate
2A9B	DHP	7	119	123	0.24	0.26	12	44	100	7	Gave larvae
3322	DHP	9	124	128	0.24	0.26	10.5	22	100	2	Gave larvae
4249	DHP	8		126		0.25	12.5	18	50	1	Gave larvae
4AF0	DHP	10		129		0.23	11	56	100	5	Gave larvae
CFC9	DHP	8	117	122	0.23	0.25	13	80	20,000	20	Gave larvae
D3F5	DHP	8	115	129	0.23	0.25	13.25	66	100	4	Gave larvae
DC03	DHP	9	122	132	0.22	0.25	11	92	500	12	Gave larvae
63E1	DHP	8	124	125	0.27	0.28					Died after DHP
D8FD	DHP	10	111	118	0.25	0.26					Died after DHP
242B	P	12	123	133	0.25	0.27	11	88	200	6	Gave larvae
32CC	P	8	124	131	0.26	0.26	12	92	30,000	21	Gave larvae
5308	P	8	125	132	0.23	0.25	11	68	300	2	Gave larvae
568F	P	8		127		0.29	11.25	87	45,000	6	Gave larvae
CFA5	P	11	111	118	0.23	0.24	14	23	20	2	Gave larvae
D732	P	9	118	125	0.23	0.25	11.25	83	1,000	6	Gave larvae
2109	P	9	114	117	0.24	0.26	17	1			No embryos
3E9A	P	8	119	120	0.26	0.27					Died after P
BDF3	P	9	112	111	0.25	0.24					Died after P

Tag: code of the female, Injection: type of injection that the female received, Nb: number of weekly injections to reach maturation, BWI_1_: body weight index (BWI) at booster moment, BWI_2_: BWI at DHP/P injection, BGI_1_: body girth index (BGI) at booster moment, BGI_2_: BGI at DHP/P injection, t(h): hours after DHP/P injection, floaters: percentage of floating eggs 1 h after fertilization, Larvae nr.: number of larvae that hatched, Survival: number of days that the larvae survived after hatching.

#### 3.2.2 RNA sequencing

36,061 transcripts that were associated with *A. anguilla* genes in NCBI were detected when comparing: 1) eggs stripped after DHP injection and eggs stripped after P injection; 2) oocytes sampled at the time of DHP injection and eggs stripped after DHP injection; 3) oocytes sampled at the time of P injection and eggs stripped after P injection. When comparing eggs that were stripped after either DHP or P injection, only one gene (*glutathione S-transferase Mu 3-like*) was significantly different and upregulated in eggs stripped after P injection (*p* < 0.01; Supplementary Table S1). The comparison between oocytes sampled at the time of DHP injection and eggs stripped after DHP injection yielded 1,710 differentially expressed genes (DEGs) (*p* < 0.01; Supplementary Table S2). While 1,599 genes of these 1,710 DEGs were shared with the comparison between oocytes sampled at the time of P injection and eggs stripped after P injection, 111 genes were not (*p* < 0.01). The comparison between oocytes sampled at the time of P injection with eggs stripped after P injection yielded 5,074 DEGs (*p* < 0.01; Supplementary Table S3). As stated, 1,599 genes of these 5,074 DEGs were shared with the comparison between oocytes sampled at the time of DHP injection and eggs stripped after DHP injection, 3,475 genes were not (*p* < 0.01).

Among the 1,710 DEGs that were differentially expressed between oocytes sampled at the time of DHP injection and eggs stripped after DHP injection (Supplementary Table S2), most genes that were targeted *in vitro* were found (Table 1). Alike the *in vitro* results (Figure 4), the *luteinizing hormone/choriogonadotropin receptor, progesterone receptor* and *androgen receptor-like* were downregulated between oocytes sampled at the time of DHP injection and eggs stripped after DHP injection (Supplementary Table S2). Alike the *in vitro* results of *ptger4b* after 18 h of incubation (Figure 4), the *prostaglandin E2 receptor EP4 subtype-like* was not differentially expressed *in vivo* (Supplementary Table S2). In contrast to the *in vitro* results (Figure 4), the *follicle-stimulating hormone receptor* was downregulated *in vivo* (Supplementary Table S2); probably due to its transient expression (Figure 4). The same results were observed when comparing the *in vitro* (Figure 4) and *in vivo* (Supplementary Table S3) results following P treatment.

On basis of fold change magnitude, the top 200 genes of the 1) 1,710 DEGs that were yielded from the comparison between oocytes sampled at the time of DHP injection and eggs stripped after DHP injection and 2) 5,074 DEGs that were yielded from the comparison between oocytes sampled at the time of P injection and eggs stripped after P injection were categorized into groups according to their biological functions to understand what happens between the injection moment and egg release (Table 4). Among these DEGs were several genes that were involved in apoptosis, cell adhesion, cell cycle, cytoskeleton organization, extracellular matrix organization, transport, lipid metabolism, muscle contraction, steroid signalling pathway and steroidogenesis. Other genes, presented in Supplementary Table S4, were involved in respiration, mRNA processing/splicing, transcription, translation and ubiquitination.

**TABLE 4:** Genes that were differentially regulated between oocytes prior the final injection and stripped eggs with FC (P): fold change resulting from the comparison between oocytes sampled prior to P injection and eggs stripped after P injection; FC (DHP): fold change resulting from the comparison between oocytes prior the DHP injection and eggs stripped after DHP injection. The * represents the leveling.

*Apoptosis	FC (P)	FC (DHP)	*Lipid metabolism	FC (P)	FC (DHP)
DNA damage-inducible transcript 4-like protein	61	15	phospholipid-transporting ATPase ABCA1	−39	−26
serine/threonine-protein kinase 17A-like	−31	−8	lipoprotein lipase	−514	−143
*Cell adhesion			alpha-2,8-sialyltransferase 8F-like	inf	−119
catenin (cadherin-associated protein), delta 1	24	13	protein ABHD15-like	inf	−234
carcinoembryonic antigen-related cell adhesion molecule 5	−282	−152	*Muscle contraction		
cadherin-2-like	inf	−53	tropomodulin-3-like	26	11
*Cell cycle			myosin-7-like	inf	inf
cell division cycle 25B	74	48	*Steroid signalling pathway		
geminin DNA replication inhibitor	70	32	CUE domain containing 2	27	41
anillin, actin binding protein	45	27	progesterone receptor	−327	−246
cyclin A1	19	12	estrogen receptor 2b	−15	−39
cyclin-J-like	18	17	progesterone receptor-like	inf	−74
cyclin I	−55	−19	androgen receptor-like	inf	−33
*Ion, water, protein transport			*Steroidogenesis		
sorting nexin-13-like	57	16	steroid 17-alpha-hydroxylase/17,20 lyase, transcript 1	−5040	−4167
protein Hook homolog 2-like	25	27	3 beta-hydroxysteroid dehydrogenase/Delta 5-->4-isomerase-like	−135	−42
vesicle-fusing ATPase-like	24	27	hydroxysteroid (17-beta) dehydrogenase 1	inf	−258
sorting nexin-13-like	57	16	steroid 17-alpha-hydroxylase/17,20 lyase, transcript 2	inf	−36
SIL1 nucleotide exchange factor	−114	−51	*Cytoskeleton organization		
ATPase Na+/K+ transporting subunit beta 1a	−153	−89	tubulin beta chain-like	−73	−14
ammonium transporter Rh type A-like	−565	−123	tubulin alpha 5	−48	−9
4F2 cell-surface antigen heavy chain-like	−35	−9	tubulin alpha chain-like	−96	−32
claudin-19-like	inf	−145	tubulin beta chain-like	−21	−12
putative claudin-24	inf	−101	*Extracellular matrix organization		
potassium inwardly rectifying channel subfamily J member 8	inf	inf	angiopoietin-like 7	inf	inf
aquaporin-4-like	inf	−508	A disintegrin and metalloproteinase with thrombospondin motifs 5	−3232	−44
			collagen alpha-1 (XV) chain-like	−203	−84

Inf for infinite: expression in the eggs was 0. Between the injection moment and egg release, several genes were involved in apoptosis, cell adhesion, cell cycle, cytoskeleton organization, extracellular matrix organization, transport, lipid metabolism, muscle contraction, steroid signalling pathway and steroidogenesis.

The 111 DEGs that were differentially expressed between oocytes at the moment of DHP injection and eggs stripped after DHP, and different from comparing oocytes at the moment of P injection and eggs stripped after P, were categorized into groups according to their biological function to understand what DHP induced more than P between the injection and egg release moment (Supplementary Table S5). Among these DEGs were several genes involved in apoptosis and inflammation, cell adhesion, cell cycle, transport, transcription and ubiquitination.

On basis of fold change magnitude, the top 200 genes of the 3,475 DEGs that were differentially expressed between oocytes at the moment of P injection and eggs stripped after P, and different from comparing oocytes at the moment of DHP injection and eggs stripped after DHP, were categorized into groups according to their biological function to understand what P induced more than DHP between the injection and egg release moment (Supplementary Table S6). Among these DEGs were several genes that were involved in cell adhesion, cell cycle, extracellular matrix organization, inflammation, ion transport, mRNA processing/splicing, muscle contraction, mitochondrial respiration, steroidogenesis, transcription and ubiquitination.

## 4 Discussion

Since the identification of DHP as the MIH in the closely related Japanese eel *A. japonica* (Ohta et al., 1996; Adachi et al., 2003), female European eels have been injected with this steroid to induce oocyte maturation and ovulation. DHP treatment can have important disadvantages as females have to ovulate within 18 h as hatching rates and fertility decrease after this period (Ohta et al., 1996; Kagawa, 2003; Palstra and van den Thillart, 2009). P, a precursor of DHP in the steroidogenic pathway, has been found to induce oocyte maturation and ovulation in fish species belonging to the elopomorphs (Adachi et al., 2003), silurids (Upadhyaya and Haider, 1986), salmonids (Jalabert, 1976), cyprinids (Jalabert, 1976; Haider and Inbaraj, 1989) and esociformes (Jalabert, 1976). Based on the two-cell type model developed in salmonids (Nagahama, 1997), it is commonly believed that P, which is formed in the theca cells from pregnenolone, is converted into 17α-hydroxyprogesterone that crosses the basal lamina and is then converted into DHP in the granulosa cells. Assuming a similar mechanism in eels, P treatment may lead to the production of endogenous DHP by the eel’s folliculated oocytes. In this study, we compared P with DHP treatment *in vitro*, to determine dose-response effects on oocyte maturation, and *in vivo*, to determine the effects on oocyte transcriptomics. Here, we showed that the effective dose to induce oocyte maturation and ovulation was similar for both steroids. Since DHP and P work equally well on batch level *in vitro* and *in vivo*, it is possible that P acts either directly as MIH or indirectly by being converted into DHP. Further argumentation for this possibility should be provided by measurement of DHP in culture medium culture after P treatments.

### 4.1 Oocyte maturation

Both DHP and P induced GVBD *in vitro* in maturing oocytes of European eels. Ovarian pieces that consisted of folliculated and de-folliculated oocytes lacked a GV from which we deduced that GVBD had occurred at doses of 100 ng DHP (67%) or 100 ng P (63%). This result is consistent with the study of Kagawa et al. (2009) who reported that about 90% and 60% of the oocytes, that were folliculated and de-folliculated, respectively, exhibited GVBD at a dose of 100 ng DHP in the Japanese eel *A. japonica*. Oocytes from the European eel might be less sensitive for DHP than oocytes from Japanese eel since doses of 10 ng and 100 ng DHP were both equally effective in inducing GVBD in *A. japonica* (Kagawa et al., 1995; Adachi et al., 2003) while in European eel the GVBD percentage was lower at a dose of 10 ng DHP. Importantly, in this study, similar dosages of both DHP and P treatments induced GVBD *in vitro* in European eels suggesting that P could either act directly as MIH, or indirectly by being converted into DHP in European eels.

Neither DHP nor P increased the oocyte diameter *in vitro* indicating an overall absence of a hydration response. DHP induced hydration in folliculated oocytes but not in de-folliculated oocytes *in vitro* in *A. japonica* (Kagawa et al., 2009). The lack of a significant hydration response in our study might be explained by the presence of de-folliculated oocytes in the ovarian pieces. In *A. japonica*, aquaporin 1 (*aqp1b*), that is essential for mediating water uptake into eel oocytes, was detected around the large yolk mass in oocytes at the migratory nucleus stages (Kagawa et al., 2011). In European eels, hydration could (also) be mediated by aquaporin 4 (*aqp4*) as our RNAseq results showed that this gene was differentially regulated between egg and oocyte stages. Lipid diameter increased in oocytes *in vitro* treated at the highest dose of P after 18 h of incubation. In previous studies, DHP at a dose of 100 ng was found to induce lipid coalescence in *A. japonica* (Kagawa et al., 2009) suggesting that lipid coalescence is responsible for the increased lipid diameter in our study.

In this study, *ara* decreased after 18 h of incubation with either DHP or P. Our observations are in line with the study of Bobe et al. (2004) who showed that *ara* expression decreased during oocyte maturation in rainbow trout *Oncorhynchus mykiss*. Androgens such as testosterone are essential for oocyte maturation in fish (Crowder et al., 2018; Li et al., 2019). In zebrafish *D. rerio*, Crowder et al. (2018) showed that loss of the androgen receptor function disrupted oocyte maturation since ovaries from mutant females contained mostly immature and only few matured oocytes when compared to the wild type. These authors speculated that androgen-mutant females had increased estrogen levels that would delay oocyte maturation in *D. rerio*. Furthermore, androgens like testosterone have been shown to induce GVBD *in vitro* in *D. rerio* oocytes (Li et al., 2019), and also in oocytes of Japanese eel (Adachi et al., 2003). The molecular mechanism behind the effects of androgenic steroids on oocyte maturation is important but still unclear in fish and has not yet been investigated in European eels.

### 4.2 Ovulation

Loss of *pgr* expression in fish (Tang et al., 2016; Liu et al., 2018), but also in mice (Robker et al., 2009), causes anovulation which demonstrates the conserved function of the Pgr in ovulation. Recently, we have demonstrated that recombinant Lh induced *pgr* expression *in vitro* in oocytes of European eels (Jéhannet et al., 2023). In that study, we suggested that Lh is preparing the oocyte for ovulation by increasing the oocyte competence to DHP. Our findings support this hypothesis since both progestin receptors (*pgr1* and *pgr2*) were downregulated by both DHP and P. Similar observations were reported, *in vitro* and *in vivo*, in zebrafish *D. rerio* (Liu et al., 2018). In teleost fish, both DHP and P can bind to the Pgr to induce ovulation (Todo et al., 2000; Hanna et al., 2010) although it has been suggested that DHP is the native ligand for the Pgr in *A. japonica* since it was the most effective steroid in both binding activity and transactivation (Todo et al., 2000).

Prostaglandins, PGE_2_ and PGF_2α_, are involved in the ovulation process of eels (Kagawa et al., 2009). Prostaglandins are essential for follicle rupture by causing actin cytoskeleton rearrangement in the follicle cells (for review see Takahashi et al., 2019). In agreement with the notion that Ptger4b is important for ovulation in zebrafish *D. rerio* (Tang et al., 2016) and medaka *O. latipes* (Hagiwara et al., 2014), our study supports that Ptger4b is also essential for ovulation in European eels. Both DHP and P treatment increased Ptger4b mRNA levels *in vitro* after incubating the maturing oocytes for 12 h which is also the moment that females release eggs *in vivo* (11–14 h). The observed changes in *ptger4b* expression are in line with the work of Baker and Van der Kraak (2019) who reported that *ptger4b* mRNA levels were upregulated in zebrafish *D. rerio* full-grown follicles treated with DHP. In medaka *O. latipes*, a model for *ptger4b* expression in preovulatory follicles destined to undergo ovulation, has been proposed by Hagiwara et al. (2014). This model shows that, following the binding of DHP to the Pgr, the activated Pgr functions as a critical transcription factor for *ptger4b* gene expression. When considering the temporal expression of the *pgr* and *ptger4b* in maturing eel oocytes and the work of Hagiwara et al. (2014), we can assume that Pgr that is produced after the Lh surge can bind MIH in order to increase *ptger4b* expression in European eels. In this study, *fshr* expression surprisingly followed the same expression pattern as the *ptger4b* expression, when oocytes were incubated with either DHP or P, indicating a role of the Fshr in ovulation. Our results agree with results from previous studies that demonstrated that *fshr* expression peaked just before oocyte maturation and ovulation in rainbow trout (Sambroni et al., 2007). Although the transcripts of *lhcgr1* decreased over time, it is possible that *lhcgr1* expression is stimulated by Lh. One day prior to the *in vitro* experiment, females were injected with CPE (that mostly contains Lh, see Minegeshi et al., 2012) to boost oocyte maturation. While the measurement at the start of the incubation might still reflect the positive influence of Lh on *lhcgr1*, the drop in *lhcgr1* expression may be explained by the lack of Lh in the *in vitro* environment. Interestingly, low doses of DHP (1 and 10 ng mL^−1^) and P (10 ng mL^−1^) increased *lhcgr1* expression when compared to the controls. These results may suggest that MIH plays a role in maintaining *lhcgr1* expression in European eels. In order to gain more insight into the roles of the Fshr and Lhr, future studies should aim to investigate the gene and protein expression levels of *fshr* and *lhrcg1* in the period before ovulation in CPE-stimulated European eel *A. anguilla*.

### 4.3 Transcriptomic signatures for oocyte maturation and ovulation

Our transcriptomic study shows that thousands of genes were differentially expressed between the time of spawning induction and egg release. In eggs of pelagophil species, the yolk that is cleaved into free amino acids and inorganic ions increase the osmotic pressure of the oocyte to allow water influx (Greeley et al., 1986; Lubzens et al., 2010). This hydration response is consistent with our transcriptomics results showing that the expression of numerous genes related to water and ion transport changed. Although the mechanism of transport and accumulation of ions remain largely unknown in fish, intracellular channels and ATPases are used to transport ions into the oocyte (reviewed by Lubzens et al., 2010). As expected, numerous genes associated with the steroid signalling pathway and steroidogenesis changed; results that are in line with previous studies in fish (Nagahama and Yamashita, 2008; Liu et al., 2017). Many transcripts related to the cell cycle (e.g., *cyclins*) also changed between the time of spawning induction and egg release. Once the MIH signal is received on the oocyte membrane and transduced into the ooplasm, cyclins are synthetized by translational activation to allow the formation of the MPF that induces meotic maturation (in goldfish *Carassius auratus;* reviewed by Nagahama and Yamashita, 2008). Several transcripts associated with apoptosis, cell adhesion, cytoskeleton organization and extracellular matrix organization changed significantly between the time of spawning induction and egg release. Ovulation is accompanied by cytoskeleton rearrangement (Ogiwara and Takahashi, 2017), apoptosis (Crespo et al., 2015; Liu et al., 2017), cell-matrix adhesion and extracellular matrix reorganization (Bobe et al., 2004; Crespo et al., 2013; Liu et al., 2017; Takahashi et al., 2019) to allow follicle rupture in fish.

Functionally, the changes in all these pathways were equal in eggs resulting from DHP and P treatment. No obvious differences between both treatments existed supporting the view that egg quality is not impacted. Both spawning induction therapies induced the release of eggs of similar quality since only one gene (*glutathione S-transferase Mu 3-like*) out of 31,061 transcripts was differentially expressed between eggs that were released by DHP and P injected feminized eels. We have shown that the effective dose to induce oocyte maturation and ovulation was close between DHP and P *in vitro* and *in vivo*. We can therefore conclude that DHP and P work equally well as MIH, supporting the alternative hypothesis that P directly induces the last steps of oocyte development. DHP was found to be the most important MIH and P was considered a precursor in its biosynthesis in many fish species (Jalabert, 1976; Upadhyaya and Haider, 1986; Nagahama, 1987; Haider and Inbaraj, 1989; Adachi et al., 2003). By acting upstream in the steroidogenic pathway, P would stimulate the production of endogenous DHP by the folliculated oocytes. Other studies in fish have shown that P can also directly bind to its nuclear receptor Pgr (Todo et al., 2000; Hanna et al., 2010). These findings feed our alternative hypothesis which may explain why the effects between DHP and P injection were so similar in our study. In this study, we provide indirect evidence that P acts directly on oocyte maturation and ovulation. Future studies should provide direct evidence that P is not converted into DHP in European eels but binds directly to DHP’s receptor by 1) incubating ovarian tissues with labeled progesterone; 2) comparing the binding affinities of DHP and P to their nuclear receptors; 3) defoliculating oocytes to reflect the solitary action of DHP and P; 4) monitoring *in vivo* plasma measurements of both steroids.

### 4.4 Reproductive success

Both spawning induction therapies, DHP and P, led to the production of thousands of larvae in our study. One female injected with DHP gave 20,000 larvae that survived until 20 dph. Similarly, two females injected with P produced 30,000 and 45,000 larvae that survived until 21 and 6 dph, respectively. Our reproduction results provide further evidence that egg quality was similar between DHP and P injected females: 1) P induced egg release around the same time as DHP did and 2) females injected with either DHP or P produced larvae without any differences in reproductive success. Previous studies in eels showed that females that rapidly release eggs after DHP injection produced better quality offspring than females that released eggs relatively late (Ohta et al., 1996; Palstra and van den Thillart, 2009). Like wild females, also feminized eels did not show a difference in timing of egg release between DHP and P treated individuals. However, feminized eels spawned eggs later than the wild ones and egg quality was lower in feminized eels. Also mortality following the final injection with either DHP or P was higher in feminized eels than in wild eels (Feminized: 45%; Wild: 22%).

## 5 Conclusion

In European eels, both DHP and P were able to induce GVBD *in vitro*. The temporal expression patterns of marker genes involved in oocyte maturation (*ara*) and ovulation (*pgr1*, *pgr2*, *lhcgr1*, *ptger4b*, *fshr*) were similar between DHP and P treatments. The lack of differences between DHP and P treatment was further confirmed *in vivo*. Based on the RNAseq results, only one gene out of 31,061 transcripts was differentially expressed in eggs after either DHP or P treatment. Both treatments led to the release of eggs of comparable quality that were equally able to produce larvae without any differences in reproductive success. Therefore, it can be concluded that DHP and P work equally well *in vitro* and *in vivo* as MIH. Future studies should investigate whether P either acts directly as MIH, or indirectly by being converted into DHP. Considering the equal competence, P is more attractive to apply as the price is 3,000 times lower than the price of DHP.

## Data Availability

The datasets presented in this study can be found in online repositories. The names of the repository/repositories and accession number(s) can be found below: GSE218444 (GEO).
