# Supplementary material for: The induction of oocyte maturation and ovulation in the European eel (Anguilla anguilla): in vitro and in vivo comparison of progesterone with 17α,20β-dihydroxy-4-pregnen-3-one
Source: Front Physiol. 2023 Aug 8;14:1207542. doi: 10.3389/fphys.2023.1207542 (PMC10442833; doi:10.3389/fphys.2023.1207542)
Supplement: Supplementary file 2 [file DataSheet1.docx]

Supplementary Material


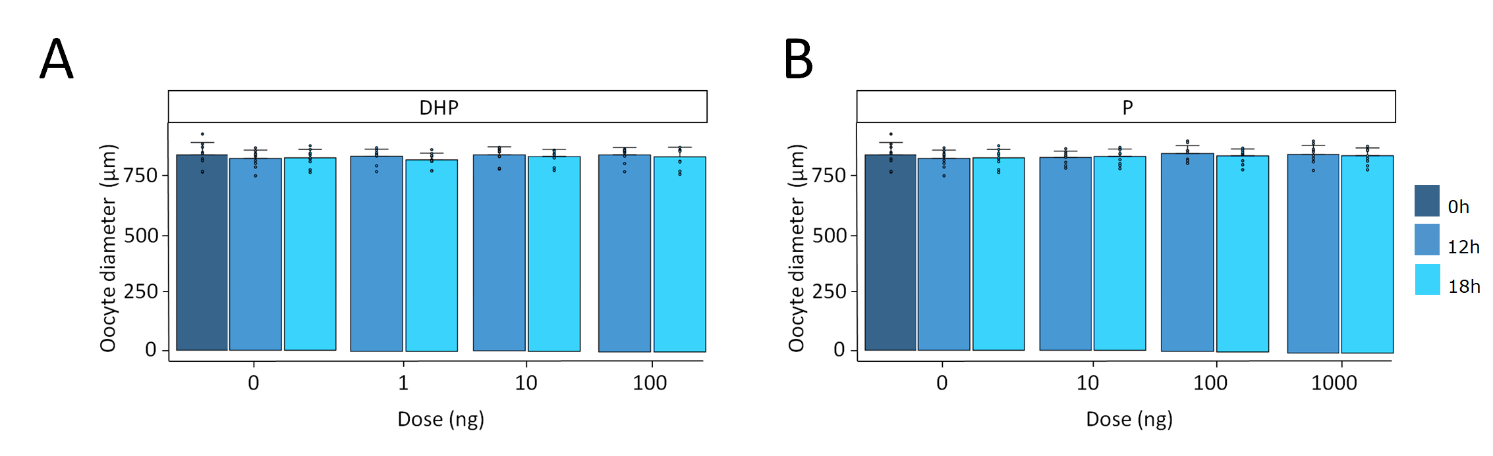


**Supplementary Figure 1.** Oocyte diameter when treated with various dosages of (**A**) DHP (0, 1, 10, 100 ng mL^-1^) and (**B**) P (0, 10, 100, 1000 ng mL^-1^) at the start of incubation (dark blue), and after 12 (mild blue) and 18 (light blue) hours of incubation *in vitro*. Measurements were compared between timepoints and doses for DHP and P. Oocyte diameter did not change with time and treatment. Data are displayed as barplots with averages ± standard deviation and individual datapoints as circles. Data are based on oocytes originating from N=10 eels.

**
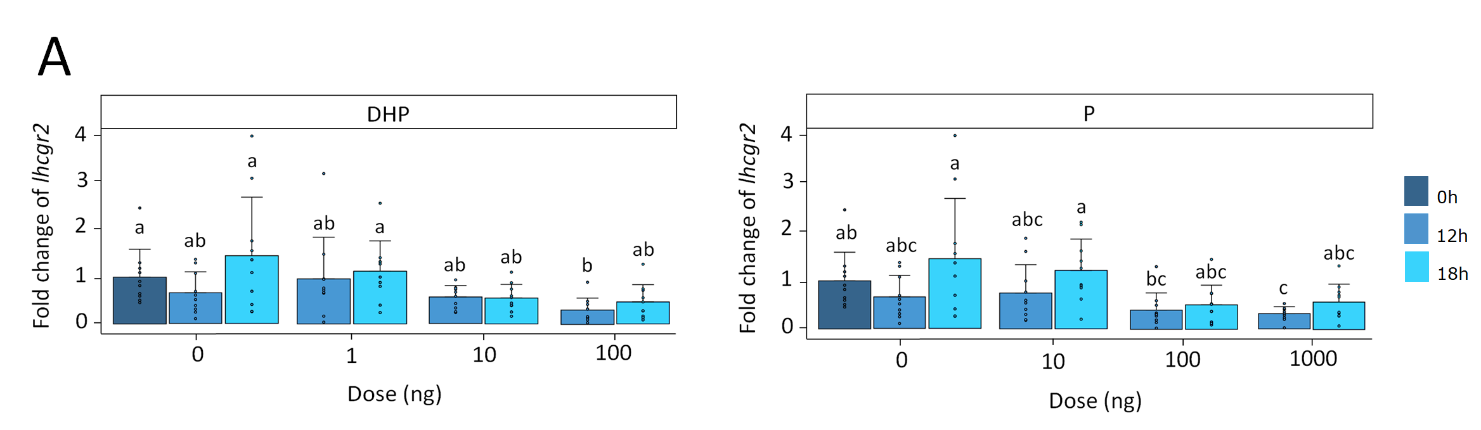
Supplementary Figure 2.** Expression of the luteinizing hormone receptors *lhcgr2* when treated with various dosages of (**A**) DHP (0, 1, 10, 100 ng mL^-1^) and (**B**) P (0, 10, 100, 1000 ng mL^-1^) at the start of incubation (dark blue), and after 12 (mild blue) and 18 (light blue) hours of incubation *in vitro*. Receptor expression was normalized to the 0 h control and expressed as fold change. Expression was compared between timepoints and doses for DHP and P. Receptor expression was normalized to the 0 h control and expressed as fold change. Expression was compared between timepoints and dosages for P and DHP. Significant differences were detected but differences were not correlated with time and treatment. Bars with no overlap in letters are significantly different (P<0.05). Data are displayed as barplots with averages ± standard deviation and individual datapoints as circles. Data are based on oocytes originating from N=10 eels.
